# Hierarchical regulation of *Burkholderia glumae* type III secretion system by GluR response regulator and Lon protease

**DOI:** 10.1111/mpp.13241

**Published:** 2022-06-19

**Authors:** Joan Marunga, Yongsung Kang, Eunhye Goo, Ingyu Hwang

**Affiliations:** ^1^ Department of Agricultural Biotechnology Seoul National University Seoul Republic of Korea; ^2^ Research Institute of Agriculture and Life Sciences Seoul National University Seoul Republic of Korea

**Keywords:** *Burkholderia glumae*, Lon protease, two‐component system, type III secretion system, virulence

## Abstract

Expression of type III secretion system (T3SS) genes, which are important for the virulence of phytopathogenic bacteria, is induced in the plant apoplastic environment or artificially amended growth conditions. Wild‐type *Burkholderia glumae* BGR1, which causes rice panicle blight, induced a hypersensitive response (HR) in tobacco plants, whereas the T3SS genes were not significantly expressed in the commonly used *hrp* induction medium. T3SS gene expression in *B. glumae* was dependent on HrpB, a well known T3SS gene transcriptional regulator. Here, we report a stepwise mechanism of T3SS gene regulation by the GluR response regulator and Lon protease in addition to HrpB‐mediated control of T3SS genes in *B. glumae*. The *gluR* mutant showed no HR in tobacco plants and exhibited attenuated virulence in rice plants. GluR directly activated *hrpB* expression, indicating that *hrpB* belongs to the GluR regulon. The *lon* mutation allowed high expression of the T3SS genes in nutrient‐rich media. Lon directly activated *gluR* expression but repressed *hrpB* expression, indicating that Lon acts as a regulator rather than a protease. However, the *lon* mutant failed to induce an HR and virulence, suggesting that Lon not only acts as a negative regulator, but also has an essential, yet to be determined role for T3SS. Our results demonstrate the involvement of the two‐component system response regulator GluR and Lon in T3SS gene regulation, providing new insight into the complex interplay mechanisms of regulators involved in T3SS gene expression in bacteria–plant interactions.

## Graphical abstract

The involvement of the TCS response regulator GluR and Lon in T3SS gene regulation hierarchically provides new insight into the complex interplay mechanisms of regulators involved in bacteria–plant interactions.

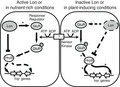

## 1 INTRODUCTION

The type III secretion system (T3SS) is a key virulence trait of many pathogenic bacteria and is highly conserved (Deng et al., 2017). The T3SS, which is encoded by a group of hypersensitive response and pathogenicity (*hrp*) genes, serves as a conduit for translocating a variety of effector proteins into plant cells, resulting in the induction of either a hypersensitive response (HR) in resistant hosts or in nonhost plants or pathogenicity in susceptible hosts (Alfano & Collmer, 1997; Balint‐Kurti, 2019; Stam et al., 2014). Expression of T3SS genes is repressed under nutrient‐rich conditions and is only induced under artificially amended conditions or in planta (Xie et al., 2019). Therefore, the T3SS is a sophisticated system with a very complex regulatory network. Assembly of the core components of T3SSs, needle length, substrate recruitment and secretion, and delivery of effector proteins are coordinately regulated in different bacterial pathogens (Deng et al., 2017). The expression of T3SS genes is transcriptionally regulated by diverse transcriptional regulators and strictly controlled by various external and host factors, such as temperature, pH, oxygen availability, and host‐derived molecules (Hutcheson et al., 2001; Ortiz‐Martin, Thwaites, Macho, et al., 2010; Ortiz‐Martin, Thwaites, Mansfield, et al., 2010; Xie et al., 2019).

Two‐component systems (TCSs) are involved in sensing T3SS‐inducing conditions and regulating *hrp* genes in the early stage of invasion (Xie et al., 2019). In *Pseudomonas syringae*, three TCSs, RhpRS, CvsRS, and GacAS, directly regulate the *hrpRS*–*hrpL*–T3SS cascade depending on the external nutrient conditions such as acetate or Ca^2+^ concentrations (Xie et al., 2019). Conversely, RhpR phosphorylated by RhpS or acetyl phosphate activates gene expression of Lon protease to result in degradation of HrpR, a transcriptional activator of *hrpL* in *P. syringae* (Zhou et al., 2016).

Lon protease has been reported to play a negative role in T3SS gene regulation via degradation of the T3SS transcriptional regulator in *Xanthomonas citri* as well as *P. syringae*. In *X. citri*, Lon regulates the T3SS through proteolysis of HrpG depending on host‐induced phosphorylation (Zhou et al., 2018). Phosphorylated Lon becomes inactive as a protease, resulting in the induction of the T3SS by HrpG‐mediated regulation in *X. citri* (Zhou et al., 2018). In addition to the negative role of Lon in T3SS gene expression, Lon together with ClpXP induces the T3SS by cleaving YmoA, a repressor protein of the T3SS gene in *Yersinia pestis* (Jackson et al., 2004). Lon is also involved in the modulation of effector protein secretion as well as the assembly of the T3SS in *P. syringae*. The half‐lives of several effectors such as AvrPto, HopPtoM, and HopPsyA are substantially higher in the *lon* mutant, suggesting rate‐limiting effector secretion via Lon‐associated degradation in *P. syringae* (Losada & Hutcheson, 2005).

We studied the pathogenic aspects of *Burkholderia glumae*, the causal agent of rice panicle blight, including toxoflavin and oxalate biosynthesis, quorum sensing (QS), QS‐dependent motility and flagellar morphogenesis, and pellicle formation (Goo et al., 2012; Jang et al., 2014; Kim et al., 2004, 2007; Kwak et al., 2020). This bacterium also relies on the T3SS for the successful infection of rice plants (Kang et al., 2008). However, the T3SS genes are not expressed in *hrp*‐inducing conditions while being activated by HrpB, a major transcriptional activator of *hrp* gene expression (Kang et al., 2008). In this study, we found that *hrp* genes are highly expressed in the *lon* mutant and that the TCS response regulator GluR plays roles in *hrp* gene expression. These results allowed us to elucidate the regulatory networks mediated by HrpB, Lon, and GluR for *hrp* gene expression in *B. glumae*. We found that Lon protease acts as a regulator to activate *gluR* expression but repress *hrpB* expression, and GluR subsequently activates the expression of *hrpB*. These findings highlight another case of Lon acting as a transcriptional regulator rather than an ATP‐dependent protease. Our study demonstrates that the interplay of GluR and Lon along with the known regulator HrpB in controlling the expression of T3SS genes is critical for virulence in *B. glumae*.

## 2 RESULTS

### 2.1 GluR was required for HR induction and full virulence of *B. glumae*

**FIGURE 1:**
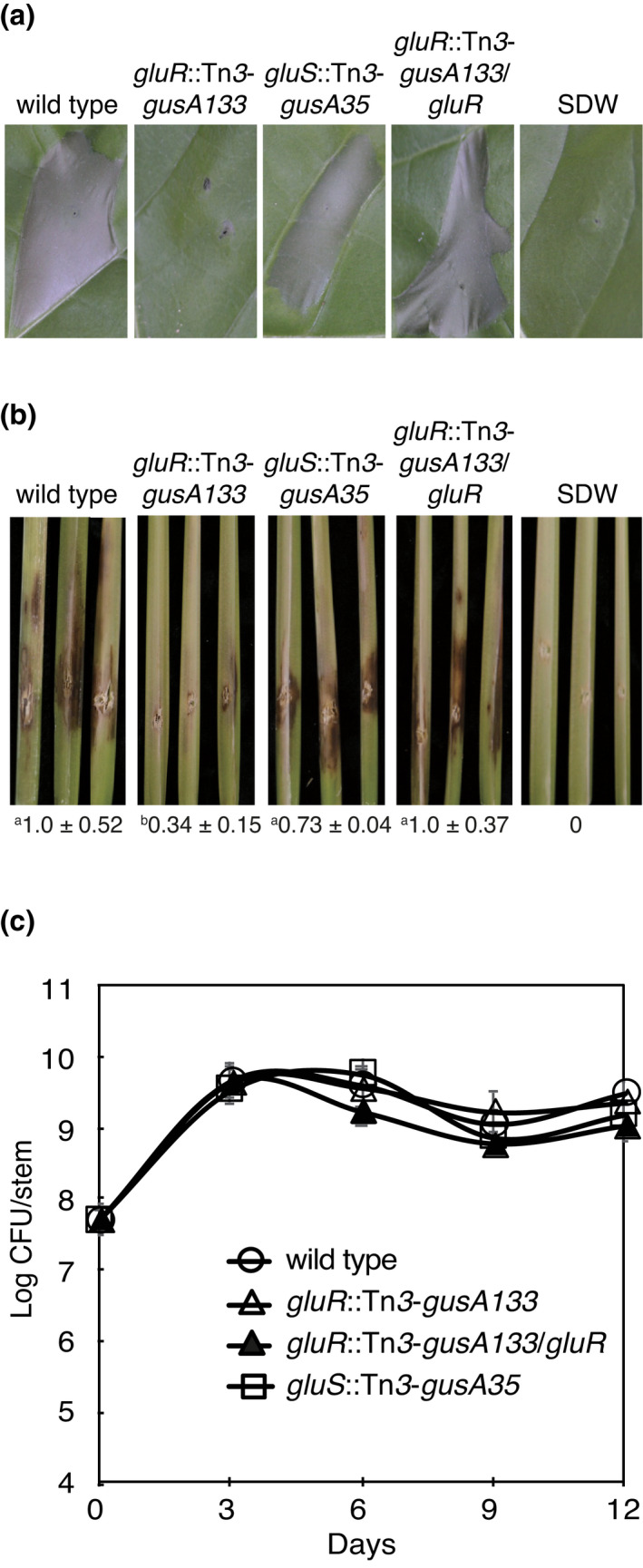
The response regulator GluR positively regulates the virulence of *Burkholderia glumae* BGR1. (a) Nonhost tobacco was inoculated with wild‐type BGR1, the *gluR* mutant (*gluR*::Tn*3*‐*gusA133*, BGLUR133), the *gluS* mutant (*gluS*::Tn*3*‐*gusA35*, BGLUS35), and the *gluR* mutant complementation strain (*gluR*::Tn*3*‐*gusA133*/*gluR*, BGLUR133C) and photographed 1 day after inoculation. SDW denotes sterile distilled water. (b) The stems of host rice plants were inoculated with the indicated *B. glumae* strains. Disease symptoms were photographed 7 days after inoculation; the numbers below the disease symptoms are the disease index scores relative to the wild‐type values. Data represent the mean ± standard error of triplicate experiments. Superscripts (a and b) before the mean value indicate statistical significances (*p* < 0.05), *F*[8,11] = 22.047 (*p* < 0.01), based on one‐way analysis of variance followed by Tukey's correlation for multiple comparisons. (c) Colonization was evaluated every 3 days after inoculation for 12 days by counting cfu of recovered cells from the infected rice stems. Data represent the mean ± standard error of triplicate experiments.

To assess whether this GluR–GluS TCS is involved in the virulence of *B. glumae*, the *gluR* mutant, the *gluS* mutant, and wild‐type BGR1 were infiltrated into tobacco leaves and injected into the stems of rice plants. The *gluS* mutant and wild‐type BGR1 induced an HR, while the *gluR* mutant failed to do so (Figure 1a). The *gluR* mutant showed significantly reduced disease symptoms with an index of 0.34 ± 0.15 compared to wild‐type BGR1 with a disease index of 1.0 ± 0.52 (Figure 1b). The complementation strain of the *gluR* mutant restored the HR and virulence as observed in wild‐type BGR1 (Figure 1a,b). The disease index of the *gluS* mutant (0.73 ± 0.04) was comparable to that of wild‐type BGR1 (Figure 1b), consistent with our previous findings that polycistronic *gluR* and *gluS* are not a functional pair (Marunga et al., 2021a). The viable cell numbers of wild‐type BGR1, the *gluR* mutant, and the *gluS* mutant were similar for the 12 days after inoculation, indicating that mutations in *gluR* or *gluS* did not affect the colonization ability of *B. glumae* (Figure 1c). Both the *gluR* and the *gluS* mutants produced toxoflavin and QS signals at the same levels as wild‐type BGR1 (Figure S1).

### 2.2 Mutation of *gluR* halted T3SS gene induction

**FIGURE 2:**
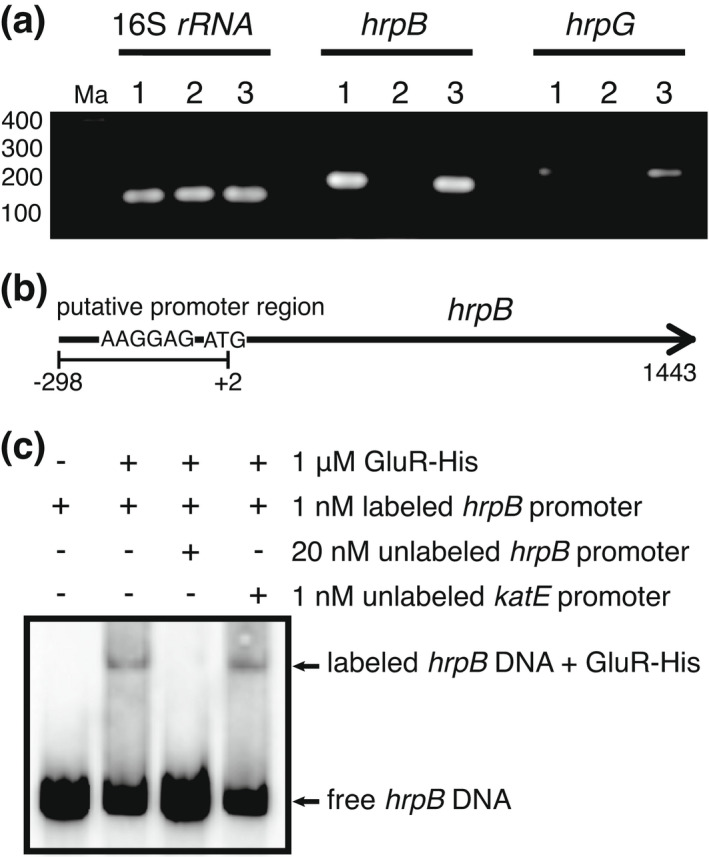
GluR positively regulates the *Burkholderia glumae* type III secretion system. (a) Reverse transcription‐PCR results showing the expression of *hrpB* and *hrpG*. 1, Wild‐type (BGR1); 2, the *gluR* mutant (BGLUR133); 3, the *gluR* mutant complementation strain (BGLUR133C). The bacterial strains were cultured in Luria‐Bertani medium supplemented with tobacco plant extracts. Ma denotes molecular markers (bp). (b) Gene map showing the putative promoter region of *hrpB* used for the electrophoretic mobility shift assay (EMSA). The arrow indicates the direction of transcription, and the bar underneath indicates the position and size of the putative promoter region. (c) EMSA showing direct binding of GluR‐His to the promoter region of the *hrpB* gene.

Given that the *gluR* mutant showed no HR induction, we wondered whether GluR is involved in the regulation of T3SS genes in *B. glumae*. Because the *hrp* genes of *B. glumae* are not expressed in Luria–Bertani (LB) or *hrp*‐inducing medium (Kang et al., 2008), we used LB supplemented with crude extracts of tobacco leaves to confirm the control of *hrpB* and *hrpG* expression by GluR. In LB medium amended with crude extracts of tobacco leaves, the *hrpB* and *hrpG* genes were expressed in wild‐type BGR1 whereas the *gluR* mutant (BGLUR133) showed no detectable expression of these two genes (Figure 2a). Complementation of the *gluR* mutant restored the ability to induce expression of the *hrpB* and *hrpG* genes in LB medium supplemented with the plant extract (Figure 2a). To demonstrate that GluR directly controls T3SS gene expression, we performed an electrophoretic mobility shift assay (EMSA) using the putative promoter region of *hrpB* (Figure 2b) and purified His‐tagged GluR (GluR‐His). The binding of GluR‐His to the putative promoter region of *hrpB* confirmed that GluR directly controls the expression of *hrpB* in *B. glumae* (Figure 2c). The *katE* promoter served as nonspecific competitor control DNA. In the upstream region of *hrpB*, we found a conserved inverted repeat sequence, comparable to those previously proposed as potential GluR‐binding sites (Marunga et al., 2021a, 2021b; Figure S2).

### 2.3 A mutation in *lon* triggered T3SS gene expression but the *lon* mutant failed to induce an HR and disease symptoms

**FIGURE 3:**
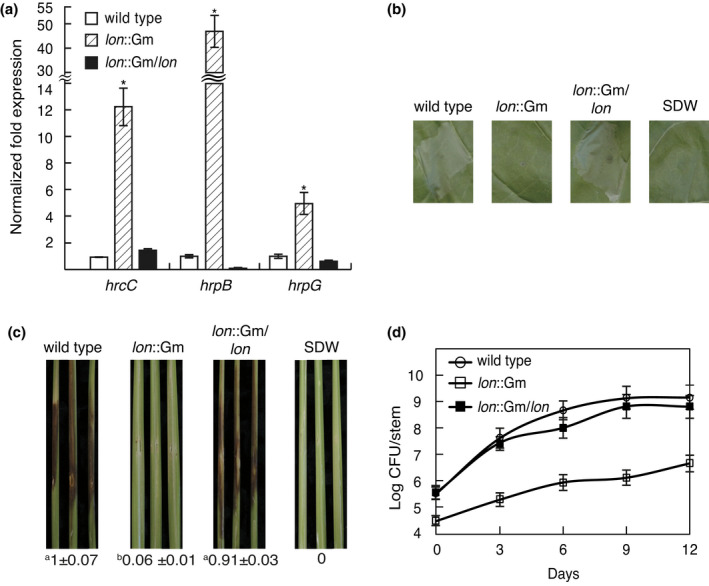
Negative regulation of *Burkholderia glumae* type III secretion system (T3SS) by Lon protease. (a) Reverse transcription‐quantitative PCR results showing the relative expression of T3SS genes (*hrcC*, *hrpB*, and *hrpG*) in the *lon* mutant (*lon*::Gm, BLONN) and the *lon* mutant complementation strain (*lon*::Gm/*lon*, BLONC) compared to that of the wild type (BGR1). *B. glumae* strains were grown in Luria‐Bertani medium. Data are mean ± standard error of triplicate experiments. Statistical analysis was performed using one‐way analysis of variance (ANOVA) followed by Tukey's test for multiple comparisons (*p < 0.001; *F*_*hrcC*_ = 92.477, *F*_*hrpB*_ = 79.132, *F*_*hrpG*_ = 58.011, *F*_0.05_[7,16] = 2.66). (b) Tobacco leaves were inoculated with *B. glumae* strains and photographed at 1 day postinoculation. SDW, sterile distilled water. (c) Disease symptoms of the indicated *B. glumae* strains in rice stems were photographed 7 days after inoculation. The numbers below the disease symptoms are the disease index scores relative to the wild‐type values. Data represent the mean ± standard error of triplicate experiments. Superscripts (a and b) before the mean value represent significant differences, based on one‐way ANOVA followed by Tukey's post hoc analysis. A value of *p* < 0.05 indicates significant differences among strains. (d) A population of *B. glumae* strains was monitored every 3 days after inoculation for 12 days by counting cfu of recovered cells from the infected rice stems. Data represent the mean ± standard error of triplicate experiments.

As we have previously reported that the T3SS genes of *B. glumae* are not expressed in artificially amended induction medium such as *hrp* induction medium (Kang et al., 2008), we expected that T3SS genes of *B. glumae* might be controlled in a different manner as compared to previously known mechanisms. During our study of the functional roles of Lon protease of *B. glumae*, we found that T3SS genes were highly expressed in the *lon* mutant in LB as assessed by RNA sequencing analysis (Table 1). We confirmed that the *hrcC*, *hrpB*, and *hrpG* genes were highly expressed in the *lon* mutant via reverse transcription‐quantitative PCR (RT‐qPCR) (Figure 3a). No expression of these three genes was observed in wild‐type BGR1, as expected (Figure 3a). However, the *lon* mutant failed to induce an HR in tobacco leaves and exhibited no disease symptoms in rice stems (Figure 3b,c). The complementation strain of the *lon* mutant exhibited a restored HR and was fully virulent, as observed in wild‐type BGR1 (Figure 3b,c). Quantification of bacterial populations in rice plants revealed that the *lon* mutant multiplied substantially more slowly than the wild type (Figure 3d). Complementation of the *lon* mutant restored virulence to wild‐type levels (Figure 3c,d). These results indicated that Lon plays a negative role in the expression of T3SS genes but is essential for HR induction and virulence.

**TABLE 1:** Type III secretion system genes are highly expressed in the *lon* mutant (BLONN) compared with the wild type (BGR1) and complemented *lon* mutant (BLONC)

Gene	Locus_ID	Reads per kilobase per million mapped reads		
		BGR1 (wild type)	BLONN (*lon*::Gm)	BLONC (*lon*::Gm/*lon*)
*hrpK*	bglu_2g02530	8	1016	12
*hrpF*	bglu_2g02520	29	4,443	37
*hrpG*	bglu_2g02500	13	171	11
*hrpW*	bglu_2g02490	5	1092	7
*hrcC*	bglu_2g02480	2	250	1
*hrpB*	bglu_2g02470	1	266	1
*hrpB1*	bglu_2g02390	2	1972	8
*hrpS*	bglu_2g02330	3	242	2
*hrpD*	bglu_2g02300	1	359	3

### 2.4 Phenotypes of the *gluR* /*lon* double mutant

**FIGURE 4:**
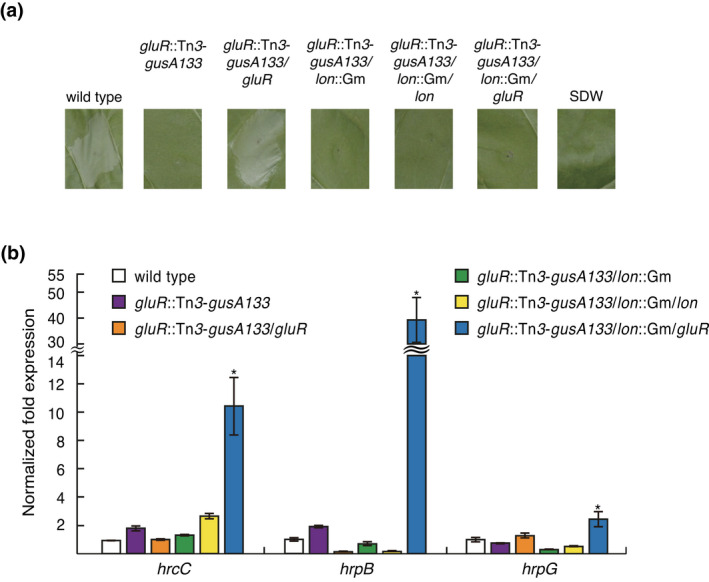
Complementation of the *lon* gene in the *gluR*/*lon* double mutant did not result in induction of a hypersensitive response and was not sufficient for type III secretion system (T3SS) gene expression. (a) Tobacco leaves were inoculated with the indicated *Burkholderia glumae* strains and photographed at 1 day postinoculation. (b) Reverse transcription‐quantitative PCR results showing the relative expression of T3SS genes (*hrcC*, *hrpB*, and *hrpG*) in the indicated *B. glumae* strains compared to that of the wild type (BGR1). The *B. glumae* strains were grown in Luria‐Bertani medium. The data are the mean ± standard error of triplicate experiments. Statistical analysis was performed using one‐way analysis of variance followed by Tukey's test for multiple comparisons (*p < 0.001; *F*_*hrcC*_ = 92.477, *F*_*hrpB*_ = 79.132, *F*_*hrpG*_ = 58.011, *F*_0.05_[7,16] = 2.66).

To determine a regulation hierarchy of T3SS genes mediated by GluR and Lon, we generated the *gluR*/*lon* double mutant and then evaluated its ability to induce an HR. The *gluR*/*lon* double mutant strains did not cause an HR (Figure 4a). Complementation of one of the two genes in the *gluR*/*lon* double mutant, resulting in *gluR* ^*−*^/*gluR* ^c^ */lon* ^*−*^ and *gluR* ^−^/*lon* ^*−*^ */lon* ^c^, showed no HR (Figure 4a). The *hrcC*, *hrpB*, and *hrpG* genes were not expressed in the *gluR* mutant and the *gluR*/*lon* double mutant; however, expression of these three genes in the *gluR*/*lon* double mutant was recovered to levels of the *lon* mutant via genetic complementation of *gluR* in trans using pBGH13 (Figure 4b). Complementation of the *lon* gene in the *gluR*/*lon* double mutant did not affect the expression of T3SS genes (Figure 4b).

### 2.5 Regulation hierarchy of T3SS genes by GluR and Lon

**FIGURE 5:**
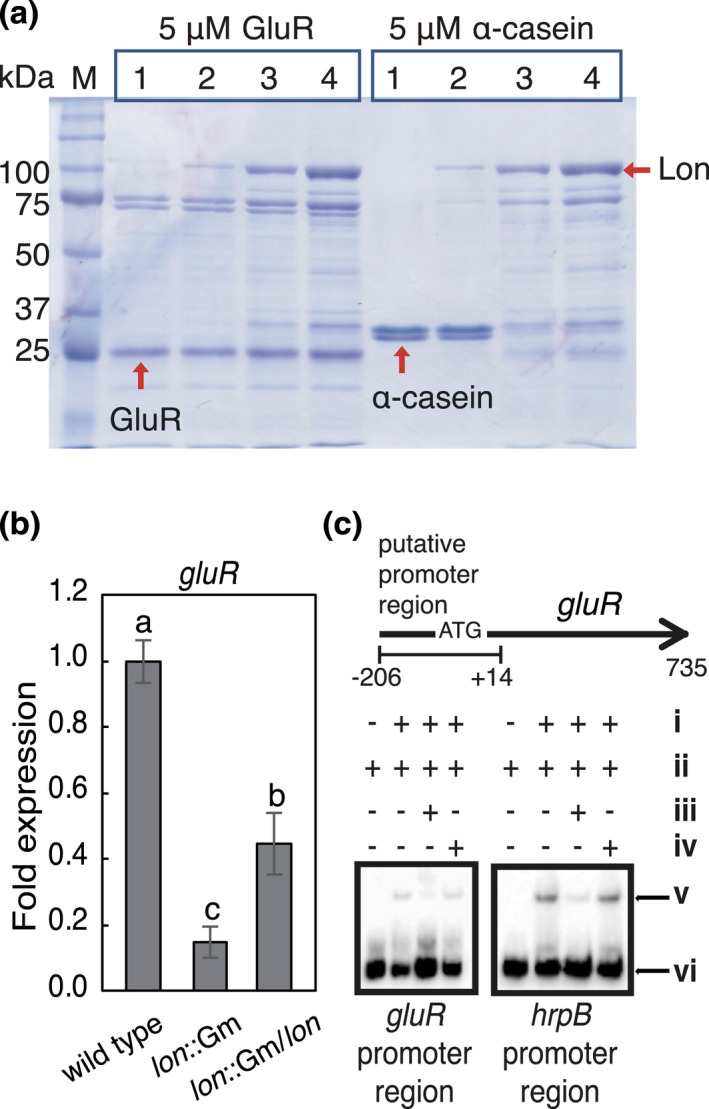
Lon protease does not degrade GluR but binds to the promoter regions of the *gluR* and *hrpB* genes. (a) In vitro protein degradation assay. Purified His‐tagged Lon samples in concentrations of 0, 1, 5, and 10 μM (reactions 1, 2, 3, and 4, respectively) were incubated with 5 μM GluR at 37°C for 1 h in the presence of 10 mM ATP. Samples were analysed by 10% SDS‐PAGE, followed by Coomassie blue staining. α‐Casein (5 μM) was used as a control substrate for Lon. The respective protein bands are labelled with arrows. M denotes molecular markers. (b) Reverse transcription‐quantitative PCR results showing the relative expression of the *gluR* gene in the *lon* mutant (*lon*::GM, BLONN) and the complementation *lon* mutant (*lon*::Gm/*lon*), BLONC) compared to that of the wild type (BGR1). Data represent the mean ± standard error of triplicate experiments. The letters (a, b, and c) above each mean indicate significant differences based on one‐way analysis of variance followed by Tukey's post hoc analysis. A value of *p* < 0.05 indicates significant differences among the strains. (c) Direct binding of His‐Lon‐His to the putative promoter regions of *gluR* and *hrpB*. (i) 0.5 μM Lon‐His; (ii) 1 nM labelled *gluR* or 0.5 nM labelled *hrpB* promoter DNA; (iii) 50 nM unlabelled *gluR* or 25 nM unlabelled *hrpB* promoter DNA; (iv) 1 nM or 0.5 nM unlabelled *katE* nonspecific competitor DNA; (v) Lon‐His + *gluR* or *hrpB* promoter DNA; (vi) free *gluR* or *hrpB* promoter DNA.

Because Lon negatively regulated the expression of *hrcC*, *hrpB*, and *hrpG* genes, we tested whether Lon functions as a protease targeting GluR or HrpB in *B. glumae*. When proteolytic activities of Lon were assessed in the in vitro degradation assay, Lon did not degrade His‐tagged GluR, whereas the known substrate of Lon protease, α‐casein, was degraded (Figure 5a). Degradation of HrpB by Lon could not be determined because most of the HrpB‐His protein formed insoluble inclusion bodies when it was overexpressed in *Escherichia coli*. Interestingly, *gluR* gene expression in the *lon* mutant showed an over fivefold reduction compared to its expression in wild‐type BGR1 (Figure 5b). The complementation strain of the *lon* mutant significantly increased the gene expression level of *gluR* (Figure 5b), suggesting that Lon positively affects the expression of *gluR* at the transcription level. To determine whether Lon directly regulates the expression of *gluR* and *hrpB*, we performed an EMSA with the putative promoter regions of *gluR* and *hrpB* and purified His‐tagged Lon (His‐Lon‐His). These results verified the direct binding of His‐Lon‐His to the putative promoter regions of *gluR* and *hrpB*, confirming the direct regulation of *gluR* and *hrpB* gene expression by Lon (Figure 5c).

## 3 DISCUSSION

Unlike other plant‐pathogenic bacteria possessing T3SSs such as *P. syringae*, T3SS expression in *B. glumae* is not induced in *hrp*‐inducing minimal medium (Kang et al., 2008). We did not understand why the *hrp* genes were not expressed in the induction medium, but studies of GluR and Lon protease in *B. glumae* revealed important clues. Two critical findings, that the *gluR* mutant failed to induce an HR and that *hrp* genes were highly expressed in the *lon* mutant, led us to elucidate the regulatory mechanisms mediated by GluR and Lon in *B. glumae*.

Recent studies have revealed how environmental signals and several TCSs play roles in the regulation of T3SS genes in pathogenic bacteria. For example, calcium, which is abundant in the plant apoplast, induces the expression of *cvsSR* in *P. syringae* pv. *tomato* DC3000, after which the response regulator CvsR binds to the *hrpRS* promoter and drives the expression of T3SS‐related genes (Fishman et al., 2018). In the DC3000 strain, the acidic amino acid utilization sensor and response regulator (AauSR) is required for maximal expression of *hrpRS* and *hrpL* in response to aspartic acid or glutamic acid, the most abundant amino acids in plants (Yan et al., 2020).

Our present study shows the first case where a TCS not clustered with T3SS genes is involved in the regulation of the *hrp* genes in *B. glumae*. The reduced virulence of the *gluR* mutant was not due to a lack of toxoflavin or QS signal production or a defect in colonization in rice (Figure S1). We believe that the reduced virulence of the *gluR* mutant is a result of previously unidentified roles of GluR besides its involvement in normal cell division and β‐lactam resistance in *B. glumae* (Marunga et al., 2021a, 2021b). The fact that the *gluS* mutant still induced an HR, unlike the *gluR* mutant, is not unusual because *gluR* and *gluS* are genetically linked but functionally unpaired for normal cell division (Marunga et al., 2021a).

*B. glumae* GluR (BGLU_1G13360) has 62.55% and 89.70% similarity to the TCS OmpR‐type response regulator of *P. syringae* (accession number AAY35331.1) and *Ralstonia solanacearum* (WP_020748442.1), respectively. GluS (BGLU_1G13350) exhibited 33.82% and 28.92% identity to the osmolarity sensor protein EnvZ of *P. syringae* (AAY35332.1) and *R. solanacearum* (CBJ40873.1), respectively. The TCS response regulator and sensor kinase of *X. oryzae* and *X. citri* showed 44.81% (WP_165480608.1) and 31.41% (WP_113139168.1) identity with *B. glumae* GluR and 46.03% (AAM35619.1) and 25.74% (AAM38857.1) identity with *B. glumae* GluS, respectively. Despite these similarities, the function of *B. glumae* GluR–GluS is quite different from those of known response regulators and sensor kinases in these plant‐pathogenic bacteria.

Regarding the loss of the HR phenotype of the *gluR* mutant, it was reasonable to hypothesize that GluR might be involved, directly or indirectly, in the control of the key transcriptional regulator HrpB in *B. glumae*. Based on EMSA results and phenotypes of the *gluR* mutant, it was clear that GluR directly activates expression of *hrpB*. However, expression levels of *hrp* genes in the wild type were not as high as we expected. This problem was solved by examining the expression level of the *hrp* genes in the *lon* mutant. Lon protease is a member of the ATPase associated with various cellular activities (AAA+) protease family and is highly conserved in prokaryotes and eukaryotes (Sauer & Baker, 2011). Lon contributes to diverse biological processes, including the heat shock response, drug resistance, DNA replication and repair, motility, and virulence factor production (Lan et al., 2007; Tsilibaris et al., 2006). Lon, which functions as a protease in *P. syringae* and *X. citri*, is also a regulator of the T3SS (Zhou et al., 2016; Zhou et al., 2018). With regard to the regulation of TCSs, there have been reports that the response regulator protein is degraded by proteases (Ogura & Tsukahara, 2010). Contrary to the roles of Lon as a protease in *P. syringae* and *X. citri*, Lon did not function as a protease to degrade GluR in vitro, but rather directly activated or repressed the expression of *gluR* and *hrpB*, respectively, in *B. glumae*. This strong evidence supports the role of Lon as a transcriptional regulator in *B. glumae*. However, no known DNA‐binding domain in Lon in *B. glumae* has been identified. A similar phenomenon was reported in *E. coli*. Lon purified from *E. coli* binds to double‐stranded DNA with no known conserved specific binding sequences and possesses no known DNA‐binding domains (Nomura et al., 2004). Moreover, Lon is a transcriptional regulator in *P. syringae*, where Lon binds directly to the promoter regions of *gacA*, *fur*, *gntR*, *clpS*, *lon*, and *glyA* to regulate various cellular functions, including motility, pyoverdine production, glucokinase activity, chaperone expression, self‐regulation of *lon*, and serine hydroxymethyltransferase activity (Hua et al., 2020). Our results are the first to demonstrate a case in which a response regulator and the T3SS are under the control of Lon at the transcriptional level.

Conventionally, transcription factors serve only as activators or repressors. However, a recent study showed that transcription factors could play a dual‐function role. For example, the QS master regulator QsmR activates the expression of the isocitrate lyase gene but represses the expression of the isocitrate dehydrogenase gene in *B. glumae* (Goo et al., 2017). Lon has also been shown to up‐regulate and down‐regulate various genes in *P. syringae* (Hua et al., 2020).

One puzzling question about the phenotype of the *lon* mutant was how the mutant lost the ability to induce an HR under high expression of *hrp* genes in the mutant. We propose two possible answers. One is that Lon might play another essential role in the proper functioning of each component in T3SS or T3SS‐dependent proteins necessary for inducing the HR, as Lon has been reported to have a chaperone‐like function (Shin et al., 2021). The other possibility is that the loss of the HR may be due to the growth defect of the *lon* mutant in vitro (Goo & Hwang, 2021). Although we do not know whether morphophysiological instability of the *lon* mutant of *B. glumae* in vitro is what actually occurs in planta, it is one plausible explanation for the loss of HR induction as well as the defect in colonization in rice.

Clearly, the lack of T3SS gene expression in *hrp* induction medium reported in previous studies was due to the functions of Lon as a repressor, as proven by genetic and biochemical evidence. However, such derepression by the null mutation in *lon* was not sufficient for the expression of T3SS genes. When T3SS gene expression was analysed in the *gluR*/*lon* double mutant, T3SS genes were not fully expressed. These results indicate that the expression of T3SS genes still requires GluR–HrpB‐mediated activation in the absence of Lon, and they also show highly coordinated regulatory systems for the expression of T3SS genes in *B. glumae*.

**FIGURE 6:**
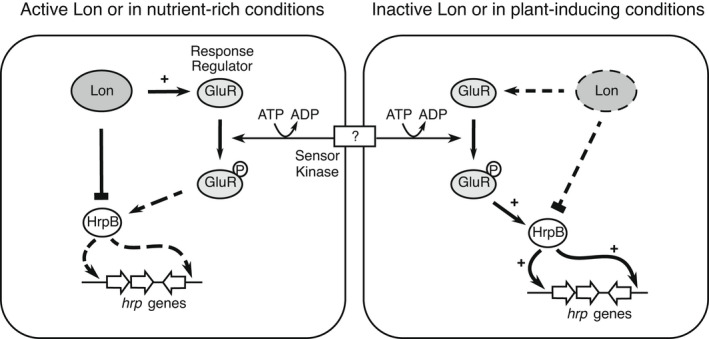
Schematic representation of the hierarchy of regulatory mechanisms of the type III secretion system (T3SS) by Lon and GluR in *Burkholderia glumae* BGR1. Regulatory pathways are proposed based on our observations. The present study showed regulation mediated by the two‐component system response regulator GluR and Lon protease in the T3SS. Lon directly represses the AraC‐type transcriptional activator *hrpB* to down‐regulate T3SS gene expression under nutrient‐rich conditions, whereas GluR positively regulates *hrpB*. Lon also activates the expression of *gluR*. + indicates activation; a flattened arrow indicates repression; p indicates phosphorylated; dashed lines indicate no activity.

In conclusion, Lon negatively regulates T3SS as a transcriptional regulator via direct binding to the promoter region of *hrpB*. In addition, Lon directly activates the expression of the *gluR* gene, but also GluR positively regulates the expression of the *hrpB* gene. Under nutrient‐rich conditions or the presence of active Lon, activation of the *hrpB* gene is not mediated by GluR (Figure 6). In the absence of Lon‐inducing conditions or under plant‐like conditions, the expression of T3SS genes is activated by GluR–HrpB (Figure 6). We believe that Lon and GluR work cooperatively to overcome host immunity and related stresses to achieve successful fitness and infection of *B. glumae*. The identification of previously unknown regulators including GluR and Lon for T3SS gene expression demonstrates that hierarchical gene regulation systems control the virulence of *B. glumae* (Figure 6) and might inform the development of various means to design effective antimicrobial drugs for the prevention of *B. glumae* spread.

## 4 EXPERIMENTAL PROCEDURES

### 4.1 Bacterial strains and growth conditions

The bacterial strains and plasmids used in this study are listed in Table S1. If not indicated otherwise, bacterial strains were grown at 37°C in LB medium containing 0.1% (wt/vol) tryptone, 0.5% (wt/vol) yeast extract, 0.5% (wt/vol) sodium chloride, and 1.5% agar when needed (Affymetrix) with appropriate antibiotics at the following concentrations: rifampicin, 100 μg/ml; ampicillin, 50 μg/ml; tetracycline, 10 μg/ml; kanamycin 50 μg/ml; gentamicin, 20 μg/ml. 5‐Bromo‐4‐chloro‐3‐indoyl‐β‐d‐galactopyranoside was used at 40 μg/ml when necessary.

### 4.2 DNA manipulation and mutagenesis

Basic DNA manipulations were done following standard protocols (Sambrook et al., 1989). DNA sequencing was performed by Macrogen, Inc. (Seoul, Korea). The genetic information and gene IDs used in this study were obtained from the *B. glumae* BGR1 genome database (GenBank accession numbers CP001503–CP001508; kropbase.snu.as.kr/cgi_bg.cg).

The TCS mutants (BGLUS35, *gluS*::Tn*3*‐*gusA35* and BGLUR133, *gluR*::Tn*3*‐*gusA133*) and the *gluR* mutant complementation strain (BLGUR133C, *gluR*::Tn*3*‐*gusA133*/*gluR*) were previously constructed (Marunga et al., 2021a). The *lon* mutant (BLONN, *lon*::Gm) and its complemented strain (BLONC, *lon*::TGm/*lon*) were previously generated (Goo & Hwang, 2021).

### 4.3 HR elicitation, virulence assay, and bacterial population in rice

Bacterial cells (1 × 10^8^ colony‐forming units [cfu]/ml) were inoculated into 4‐week‐old tobacco leaves for HR assays (Klement, 1963) and into Milyang‐23 rice plants to assess the virulence. The plants were kept in an HB‐303D‐L growth chamber (Hanbaek Scientific) for 24 h for the HR test and for 12 days for the virulence test at 30°C with 16 h light/8 h dark. The pixels of the diseased area were counted by ImageJ v. 1.53a software. The disease index was calculated by comparison with the wild‐type values as follows: disease index = disease pixels/disease pixels from the wild type. To determine the population density, the rice stems 3 cm above and below the inoculated sites were minced and ground using mortar and pestle with 1 ml sterile distilled water. Aliquots of 100 μl from each sample were serially diluted, and 10 μl each of three repeats was spotted on LB agar medium to monitor cfu at a 3‐day interval for 12 days. LB agar plates were incubated at 37°C for 24 h to allow colonies to grow. Colonies were counted under a dissecting microscope and multiplied by the appropriate dilution factor to calculate cfu/ml. The virulence and colonization assays were repeated three times with three independent replicates.

### 4.4 Toxoflavin assay

Toxoflavin was extracted from overnight cultures using chloroform as previously described (Yoneda et al., 1971). Chloroform extracts were dissolved in dimethyl sulphoxide and applied to a silica gel 60 thin layer chromatography plate (Merck). Chromatograms were developed with chloroform/methanol (95:5, vol/vol). The spots were visualized under UV light at 365 nm.

### 4.5 Autoinducer assay

The QS signal production assay was performed as previously described (Kim et al., 2004), with a few modifications. The acyl‐homoserine lactones were extracted from overnight bacterial cultures by mixing the cell‐free supernatant and ethyl acetate (1:1). The ethyl acetate extracts were evaporated using a rotary evaporator below 40°C, and the residues were reconstituted in 10 μl of dimethyl sulphoxide. A 5‐μl sample was then dropped on LB agar medium containing a *Chromobacterium violaceum* biosensor, and the plates were incubated at 28°C overnight.

### 4.6 Preparation of plant extracts

Tobacco leaves that were 4 weeks old were used to make crude plant extract. The leaves were washed under running water and dried at 37°C, and dry weight was determined before being crushed into powder. The powder was soaked in 95% methanol at a methanol:plant powder ratio of 6:1 and the mixture was incubated for 48 h at room temperature. The plant residues were filtered and methanol was removed using rotor evaporation. The residue was suspended in water and sterilized using a filter. To mimic apoplast conditions in LB medium, we used 100 mg/L crude plant extract.

### 4.7 RNA extraction and RT‐qPCR

Total RNA was isolated from *B. glumae* strains and cDNA was synthesized as previously described (Marunga et al., 2021a) using Recombinant RNasin and M‐MLV reverse transcriptase following the manufacturer's instructions (Promega). Using primers listed in Table S2, designed to amplify specific genes, transcript levels were determined using either SsoFast EvaGreen Supermix (Bio‐Rad) or the TaKaRa Taq PCR kit (Clontech) using a thermocycler (Model C1000; Bio‐Rad) at the following thermal cycling conditions: 95°C for 30 s, followed by 30 cycles of 95°C for 5 s and 60°C for 5 s. All reactions were performed in triplicate and transcript levels were normalized to the 16S rRNA gene by Bio‐Rad CFX Manager software.

### 4.8 RNA sequencing

Total RNA was extracted from *B. glumae* BGR1, BLONN (*lon*::Gm), and BLONC (*lon*::Gm/*lon*) grown in LB medium at 37°C for 10 h after subculture using RNeasy mini kits (Qiagen) following the manufacturer's protocols. Extracted total RNA was treated with RNase‐free DNase I (Ambion) to remove DNA. The quantity and quality of the total RNA were evaluated using RNA electropherograms (Agilent 2100 Bioanalyzer) and by assessing the RNA integrity number. From each sample with an RNA integrity number value greater than 8.0, 8 μg of total RNA was used as starting material and treated with the MICROBExpress mRNA Enrichment kit (Invitrogen). The resulting mRNA samples were processed for the sequencing libraries using the Illumina mRNASeq Sample Preparation kit (Illumina) following the manufacturer's protocols. One lane per sample was used for sequencing by the Illumina Genome Analyzer IIx (Illumina) to generate nondirectional, single‐ended, 36‐base‐pair reads. Quality‐filtered reads were mapped to reference genome sequences (NCBI BioProject accession: PRJNA59397 ID: 539397, http://www.ncbi.nlm.nih.gov/bioproject/59397) using the BWA package (Li & Durbin, 2009). The mRNA reads were normalized to reads per kilobase per million mapped reads (Mortazavi et al., 2008). The NCBI SRA accession number for the RNA sequencing data series of BGR1, BLONN, and BLONC is PRJNA727974.

### 4.9 EMSA

GluR‐His and His‐Lon‐His were purified as described previously (Goo & Hwang, 2021; Marunga et al., 2021a). Using primer sets hrpBp‐F/R and glurp‐F/R, listed in Table S2, putative promoter regions of the respective genes were amplified and labelled with biotin using Lightshift Chemiluminescent Electrophoretic Mobility Shift Assay Kits, as described by the manufacturer (Pierce). The EMSAs were performed as previously described (Kim et al., 2007). The putative promoter region of *katE1* (329 bp) was used as nonspecific competitor DNA, and band detection was done following previously described methods (Marunga et al., 2021a).

### 4.10 Protein in vitro degradation assay

The degradation assay was performed as previously described (Zhou et al., 2018) with a few modifications. First, 5 μM of GluR‐His was mixed with varying concentrations of His‐Lon‐His (0, 1, 5, and 10 μM) in Lon degradation buffer (10 mM ATP, 10 mM MgCl_2_, 25 mM Tris.HCl [pH 8.0], 100 mM NaCl). The reactions were incubated at 37°C for 1 h. α‐Casein (5 μM; Sigma‐Aldrich) was used as the control substrate. The reaction was stopped by adding SDS loading dye and proteins were denatured by boiling. The samples were then separated by 10% SDS‐PAGE and visualized using Coomassie blue G‐250 stain.

### 4.11 Statistical analyses

All experiments were conducted in triplicate with the respective controls. One‐way analysis of variance (ANOVA) was used followed by Tukey's honestly significant difference post hoc analysis using SPSS statistical software (v. 26; IBM Corp.) to detect significant differences where required. A *p*value of <0.05 indicates statistical significance. All figures were prepared using Illustrator software (v. 24.3; Adobe Inc.).

## AUTHOR CONTRIBUTIONS

Joan Marunga, Eunhye Goo, and Ingyu Hwang designed the experiments. Joan Marunga, Eunhye Goo, and Yongsung Kang performed the experiments. Joan Marunga, Eunhye Goo, Yongsung Kang, and Ingyu Hwang analysed the data. Joan Marunga, Eunhye Goo, and Ingyu Hwang contributed reagents/materials/analysis tools. Joan Marunga, Eunhye Goo, and Ingyu Hwang wrote the paper.

## CONFLICT OF INTEREST

The authors declare that the research was conducted in the absence of any commercial or financial relationships that could be construed as a potential conflict of interest.

## Supporting information


**Figure S1** The *gluS* and *gluR* mutants produced (a) toxoflavin and (b) autoinducers to the same levels as wild‐type BGR1. 1, BGR1; 2, *gluS*::Tn*3*‐*gusA35*; 3, *gluR*::Tn*3*‐*gusA133*.

**Figure S2** The sequences upstream of *hrpB* and *gluR* used in the electrophoretic mobility shift assay.

**Table S1** Bacterial strains that were used in this study.

**Table S2** List of primers used in this study.

## Data Availability

All data sets generated for this study are included in this article or supporting information; further inquiries can be directed to the corresponding author.
