# Supplementary material for: Hierarchical regulation of Burkholderia glumae type III secretion system by GluR response regulator and Lon protease
Source: Mol Plant Pathol. 2022 Jun 19;23(10):1461–71. doi: 10.1111/mpp.13241 (PMC9452761; doi:10.1111/mpp.13241)
Supplement: Supplementary file 1 — Figure S1 The gluS and gluR mutants produced (a) toxoflavin and (b) autoinducers to the same levels as wild‐type BGR1. 1, BGR1; 2, gluS::Tn3‐gusA35; 3, gluR::Tn3‐gusA133. [file MPP-23-1461-s001.docx]

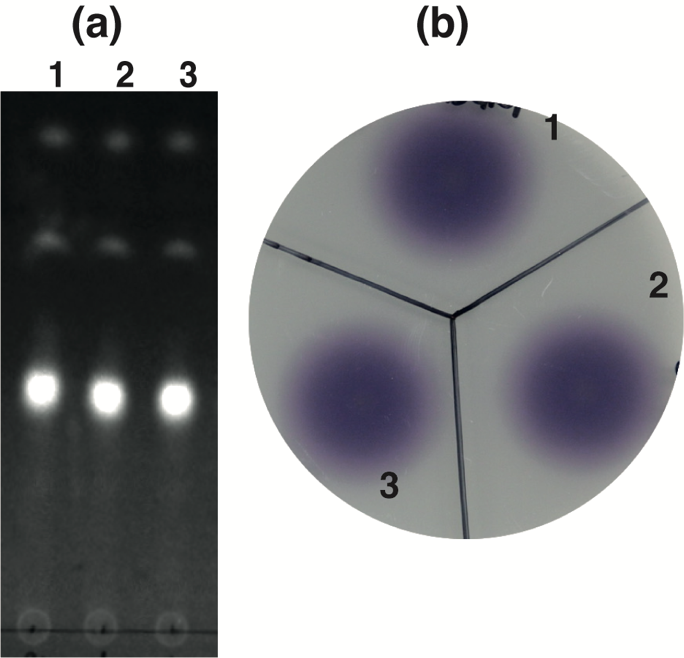


**Figure S1** The *gluS* and *gluR* mutants produced (**a**) toxoflavin and (**b**) autoinducers to the same levels as those produced by the wild type BGR1*.* 1, BGR1; 2, *gluS*::Tn*3*-*gusA35*; 3, *gluR*::Tn*3*-*gusA133*.
