# Supplementary material for: Hierarchical regulation of Burkholderia glumae type III secretion system by GluR response regulator and Lon protease
Source: Mol Plant Pathol. 2022 Jun 19;23(10):1461–71. doi: 10.1111/mpp.13241 (PMC9452761; doi:10.1111/mpp.13241)
Supplement: Supplementary file 2 — Figure S2 The sequences upstream of hrpB and gluR used in the electrophoretic mobility shift assay. [file MPP-23-1461-s003.docx]

*hrpB* promoter region

TGAGCGCGTTCACCTCGCAATTCCAGGCCTGGGCCGATGCCGCGCGCGACGGCGCCGCTCATACAAAGTGAGGTTTCGTGCGCCAAAAGTGCCGCGCGCGCGAGGCAGGCCGGCAAGAAGTTCGCCTGAGCAATCATGAGACTCGCACGAGGCCGTGCCCCGCCGTGCGCGAATCTCAAATCGGCATCATTGCTGAAGATTGCTCAGCCAAACTGAGAGTTCGAAAACGAGGCCCCGACTACATTGCTCGAACTGCGCCGCGATCCGGTGCCCAGGCAACAGCAAGGAGAGTCGCCTC**ATG**


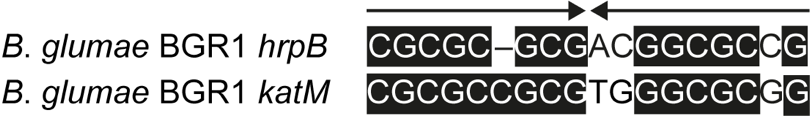


*gluR* promoter region

ACCTCTGGTACTTGAACGAACCCGGCGAATATCGGGCGAACGAACTACGAAAAATCGGGTGGGTGGAGTATCCACTGAATTTCCGCGCCAAGTAAAGGAGTGTCTAACGAGCGGCTTTACTCAACATCGCAGCACGGGTAAATTTTGTAACCGACTGTTACTCGCAAAATCCCGCGCCCGTGCGCCCCTTCGTAATCGCGATAAG**ATG**CCGCCCATGGA

**Figure S2** The sequences upstream of *hrpB* and *gluR* used in the EMSA. Sequences underlined in red are conserved inverted repeat sequences, while those underlined in black are possible ribosome binding sites. Arrows indicate the inverted repeat sequences for GluR binding with the conserved regions in the *hrpB* promoter region highlighted in black. Lon protease binds to GT-rich regions (Liu et al., 2004); conserved GT-rich regions in the *gluR* and *hrpB* promoter sequences are highlighted in yellow.

**Reference**

Liu, T., Lu, B., Lee, I., Ondrovičová, G., Kutejová, E. and Suzuki, C. K. (2004) DNA and RNA Binding by the Mitochondrial Lon Protease Is Regulated by Nucleotide and Protein Substrate. *Journal of Biological Chemistry*, 279, 13902-13910.
