# Supplementary material for: Hierarchical regulation of Burkholderia glumae type III secretion system by GluR response regulator and Lon protease
Source: Mol Plant Pathol. 2022 Jun 19;23(10):1461–71. doi: 10.1111/mpp.13241 (PMC9452761; doi:10.1111/mpp.13241)
Supplement: Supplementary file 3 — Table S1 Bacterial strains that were used in this study. [file MPP-23-1461-s002.docx]

**Table S1** Bacteria strains that were used in this study.

| Strain or plasmid | Characteristics | Source |
| --- | --- | --- |
| *Burkholderia glumae* |  |  |
| BGR1 | Wild type, Rif^R^ | (Kim et al., 2004) |
| BGLUR133 | BGR1, *gluR*::Tn*3*-*gusA133*, Km^R^ | (Marunga et al., 2021) |
| BGLUS35 | BGR1, *gluS*::Tn*3*-*gusA35*, Km^R^ | Marunga et al., 2021) |
| BGLUR133C | BGR1, *gluR*::Tn*3*-*gusA133* containing pBGH13, Km^R^, Tet^R^ | Marunga et al., 2021) |
| BLONN | BGR1, *lon*::Gm, Gm^R^ | (Goo and Hwang, 2021) |
| BLONC | BGR1, *lon*::Gm carrying pLa4, Gm^R^, Tet^R^ | (Goo and Hwang, 2021) |
| BGLURLON | BGR1, *gluR*::Tn*3*-*gusA133*, *lon*::Gm, Gm^R^, Km^R^ | This study |
| BGLURLONC | BGR1, *gluR*::Tn*3*-*gusA133*, *lon*::Gm containing pLa4, Gm^R^, Km^R,^ Tet^R^ | This study |
| BGLURCLON | BGR1, *gluR*::Tn*3*-*gusA133*, *lon*::Gm containing pBGH13, Gm^R^, Km^R,^ Tet^R^ | This study |
| *Escherichia coli* |  |  |
| DH5α | F– Φ80d*lacZ ΔM15*(*lacZYA-argF*) U169 *recA1* *endA1 hsdR17* (r_k_^+^m_k_^+^) *supE44* *thi-1 gyrA relA1* | Gibco BRL |
| BL21 (DE3) | F^–^ *ompT hsdS_B_* (rB^-^m_B_^-^) *gal dcm* (DE3) | Novagen |
| Plasmid |  |  |
| pBluescript II SK (+) | Cloning vehicle; phagemid, pUC derivative, Amp^R^ | Stratagene |
| pRK2013 | Tra^+^, ColE1 replicon, Km^R^ | (Figurski and Helinski, 1979) |
| pLAFR3 | Tra^-^, Mob^+^ RK2 replicon, Tet^R^ | (Staskawicz et al., 1987) |
| pET21b | T7 promoter-based expression vector, Amp^R^ | Novagen |
| pLa4 | 2.9 kb pLa3 fragment cloned into *Hind*III and *Eco*R1 sites in pLAFR3, Tet^R^ | (Goo and Hwang, 2021) |
| pBGH13 | 6.3 kb *Bam*H1-*Hin*dIII fragment containing *gluR* gene, Tet^R^ | Marunga et al., 2021) |
| pGluR-His | *gluR* in pRT21b, Amp^R^ | Marunga et al., 2021) |
| pLon-His | *lon* in pRT21b, Amp^R^ | This study |
| pHrpB-His | *hrpB* in pRT21b, Amp^R^ | This study |
|  |  |  |
|  |  |  |

Rif^R^rifampicin resistance; Tet^R^, tetracycline resistance; Km^R^, Kanamycin resistance; Amp^R^, ampicillin resistance; Gm^R^, Gentamicin resistance

**REFERENCES**

Figurski, D.H. and Helinski, D.R. (1979) Replication of an origin-containing derivative of plasmid RK2 dependent on a plasmid function provided in trans. *Proceedings of the National Academy of Sciences of the United States of America,* 76, 1648-1652.

Goo, E. and Hwang, I. (2021) Essential roles of Lon protease in the morphophysiological traits of the rice pathogen *Burkholderia glumae*. *PLoS ONE*, 16, e0257257.

Kim, J., Kim, J.G., Kang, Y., Jang, J.Y., Jog, G.J., Lim, J.Y. et al. (2004) Quorum sensing and the LysR‐type transcriptional activator ToxR regulate toxoflavin biosynthesis and transport in *Burkholderia glumae*. *Molecular Microbiology,* 54, 921-934.

Marunga, J., Goo, E., Kang, Y. and Hwang, I. (2021) Identification of a Genetically Linked but Functionally Independent Two-Component System Important for Cell Division of the Rice Pathogen *Burkholderia glumae*. *Frontiers in Microbiology*. 12, 700333.

Staskawicz, B., Dahlbeck, D., Keen, N. and Napoli, C. (1987) Molecular characterization of cloned avirulence genes from race 0 and race 1 of *Pseudomonas syringae* pv. glycinea. *Journal of Bacteriology,* 169, 5789-5794.
