# Supplementary material for: Hierarchical regulation of Burkholderia glumae type III secretion system by GluR response regulator and Lon protease
Source: Mol Plant Pathol. 2022 Jun 19;23(10):1461–71. doi: 10.1111/mpp.13241 (PMC9452761; doi:10.1111/mpp.13241)
Supplement: Supplementary file 4 — Table S2 List of primers used in this study. [file MPP-23-1461-s004.docx]

**Table S2** List of primers used in this study.

| **Primers** | **Sequence (5’ to 3’)** |
| --- | --- |
| 16S RNA-F | AGCCGCGGTAATACGTAGG |
| 16S RNA-R | ACTCTAGCCTGCCAGTCACC |
| HrpB-F | TGCGCCTATCTCGAACGGA |
| HrpB-R | AGAGATATTCGAGCTGGCGA |
| HrpG-F | CGATTCTCCCGTCAGTTCCA |
| HrpG-R | CCTATACGCTCGACCAGCA |
| HrcC-F | ACCAACTCGGTGCTGATCC |
| HrcC-R | AGCGTGTTGTCGTCGATCTC |
| GluR-F | CAAACACCTTCAGCACCGAG |
| GluR-R | CGGACGATTACCTGCCCAAG |
| KatE-F | AACCATGTGACGAACGACGA |
| KatE-R | TACATATCGCTGAGCGTGGC |
| hrpBp-F | TGAGCGCGTTCACCTCGCAA |
| hrpBp-R | ATGAGGCGACTCTCCTTGCT |
| gluRp-F | ACCTCTGGTACTTGAACGAAC |
| gluRp-R | TCCATGGGCGGCATCTTATCG |
| Nde-HrpB | GCCTTCGAACGCGAGCTCGCAGAGCCTCCCGCG |
| HrpB-HindIII | CGGAAGCTTGCGCTCGAGCGTCTCGGAGGGCGC |
| Nhe1_La | CCGCTAGCATGTCAGGCACCCAACTTCTC |
| La_HindIII | CCAAGCTTGTGCTTGACGACTTCCGTGGA |

F- forward primer; R – reverse primer
